# Supplementary material for: Confined Cu-OH single sites in SSZ-13 zeolite for the direct oxidation of methane to methanol
Source: Nat Commun. 2023 Nov 24;14:7705. doi: 10.1038/s41467-023-43508-4 (PMC10673993; doi:10.1038/s41467-023-43508-4)
Supplement: Supplementary file 1 — Supplementary Information [file 41467_2023_43508_MOESM1_ESM.pdf]

Supplementary Information

Confined Cu-OH Single Sites in SSZ-13 Zeolite for the Direct Oxidation of Methane to Methanol

Hailong Zhang[1,2,#], Peijie Han[1,#], Danfeng Wu[1], Congcong Du[1], Jiafei Zhao[1], Kelvin H.L. Zhang[1], Jingdong Lin[1], Shaolong Wan[1], Jianyu Huang[3], Shuai Wang[1,*], Haifeng Xiong[1,*], Yong Wang[4,*]

[1]State Key Laboratory of Physical Chemistry of Solid Surfaces, College of Chemistry and Chemical Engineering, Xiamen University, 422 South Siming Road, Xiamen 361005, China

[2]College of Carbon Neutrality Future Technology, Sichuan University, Chengdu 610064, China

[3]Clean Nano Energy Center, State Key Laboratory of Metastable Materials Science and Technology, Yanshan University, Qinhuangdao 066004, China

[4]Voiland School of Chemical Engineering and Bioengineering, Washington State University, Pullman, WA 99164, United States

TABLE OF CONTENT

Figures 3
Tables 16
Analysis and discussion 21
FTIR simulation by DFT calculation 21
H_2 temperature-programmed reduction (H_2-TPR) 21
DR UV-Vis spectroscopy 22
X-ray absorption spectroscopy (XAS) results 22
Charge density difference (CDD) analysis 23
Temperature-programmed surface reactions (TPSR) 23
Activity tests for MTM under aerobic conditions 24
In situ FTIR spectroscopy 24
In situ UV-Vis spectroscopy 25
Isotope labeling experiments 26
DFT calculation analysis 27
References: 28

Figures

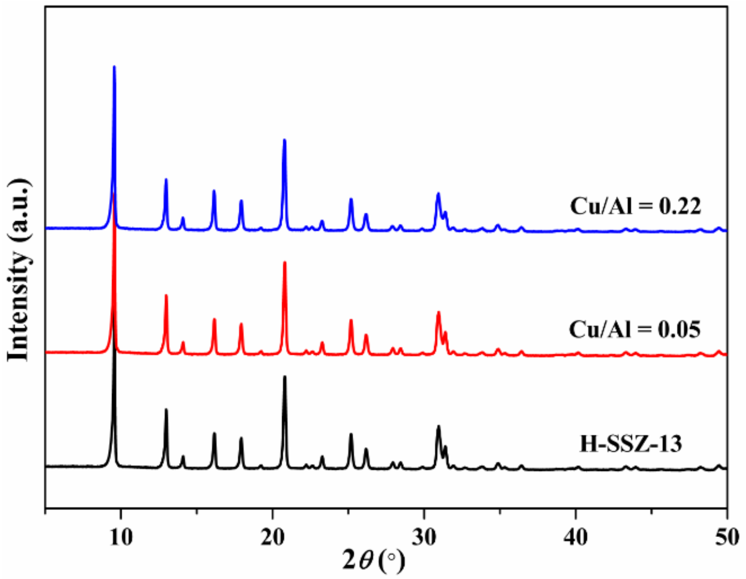

Fig. 1. XRD patterns of Cu ion-exchanged SSZ-13 zeolites. No diffraction peaks of CuO (2θ = 35.6° and 38.8°) and Cu_2O (2θ = 36.4°) were found.

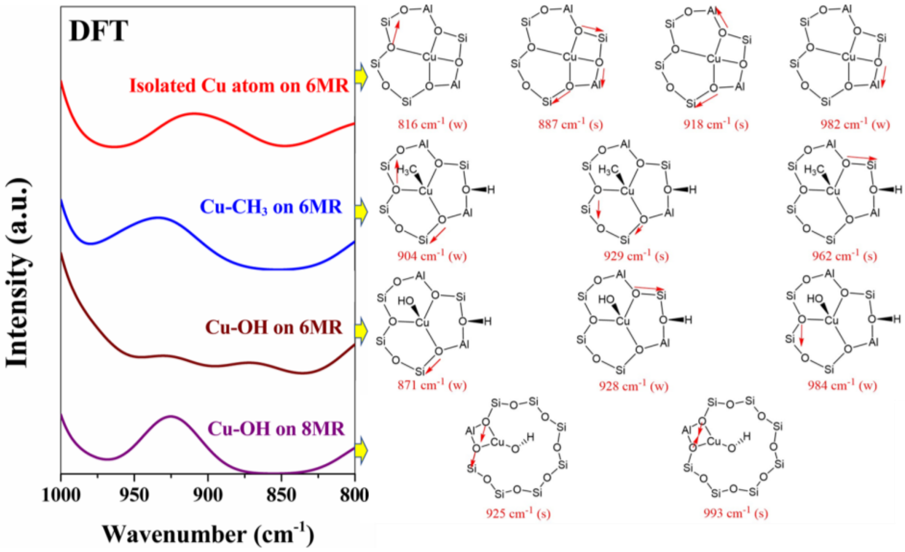

Fig. 2. DFT-calculated IR spectra belonging to different monocopper species located on 6MR and 8MR of CHA zeolite. The vibration models of copper species on 6MR and 8MR were given on the right of the figure. The weak and strong vibration intensities were denoted as “w” and “s”, respectively.

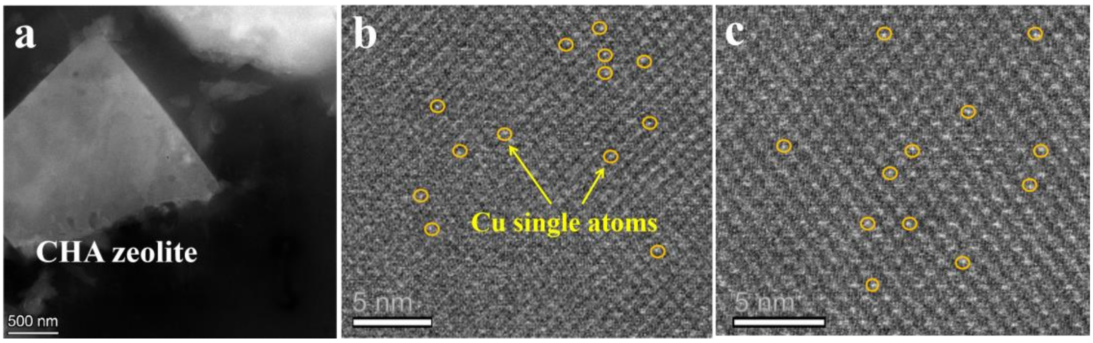

Fig. 3. HAADF-STEM images of Cu_1/SSZ-13 catalyst with Cu/Al = 0.05.

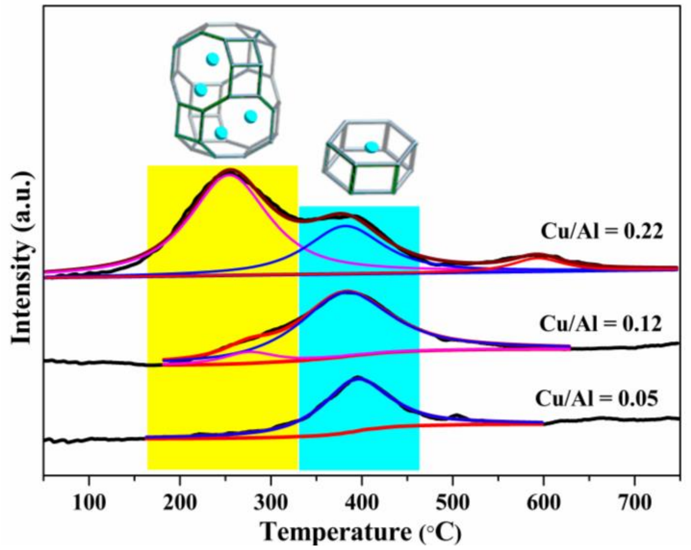

Fig. 4. H_2-TPR profiles of Cu/SSZ-13 catalysts with the Cu/Al ratio increasing from 0.05 to 0.22. Prior to the measurements, all samples were pretreated at 400 °C in the flowing helium for 30 min. Mass of sample = 100 mg; heating rate = 8 °C min^{-1}; total flowing rate = 30 ml min^{-1}.

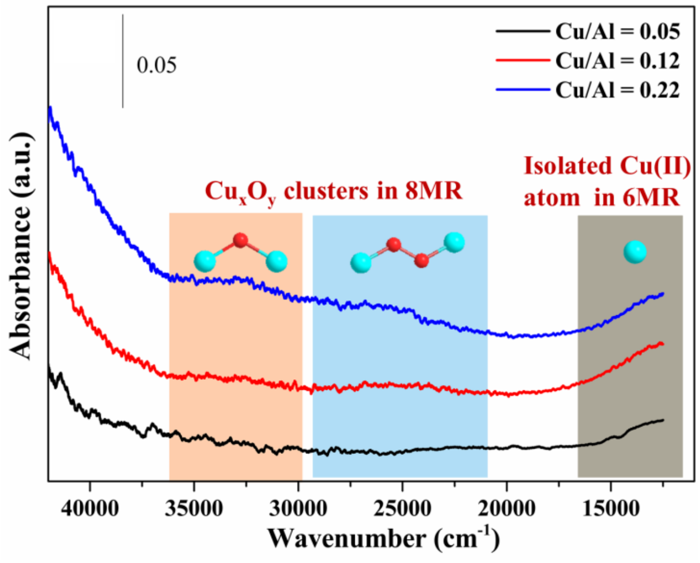

Fig. 5. DR UV-Vis spectra of Cu/SSZ-13 catalysts with the Cu/Al ratio increasing from 0.05 to 0.22. Prior to the measurements, all samples were was diluted with $BaSO_4$ powders at a mass ratio of 1/2 (catalyst/$BaSO_4$) and pretreated at 400 °C in the flowing helium for 30 min. The spectra were collected at room temperature by subtraction of $BaSO_4$ background spectrum.

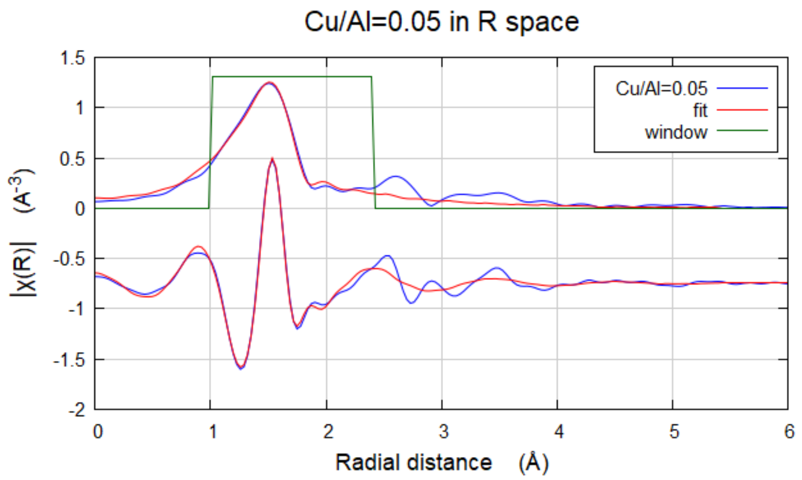

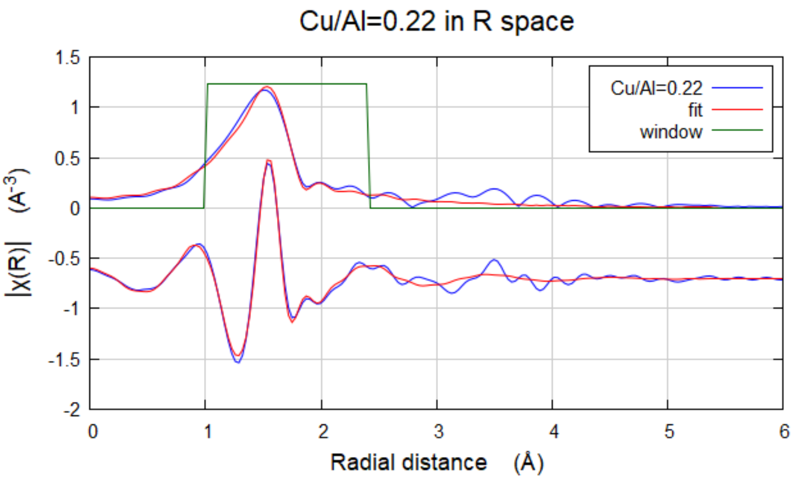

Fig. 6. EXAFS functions and the fittings of Cu/SSZ-13 catalysts with Cu/Al = 0.05 and 0.22 for the Cu K filtered k^2-weighted χ(k) and the Fourier Transform function in the k range of 3-14.3 $Å^{-1}$.

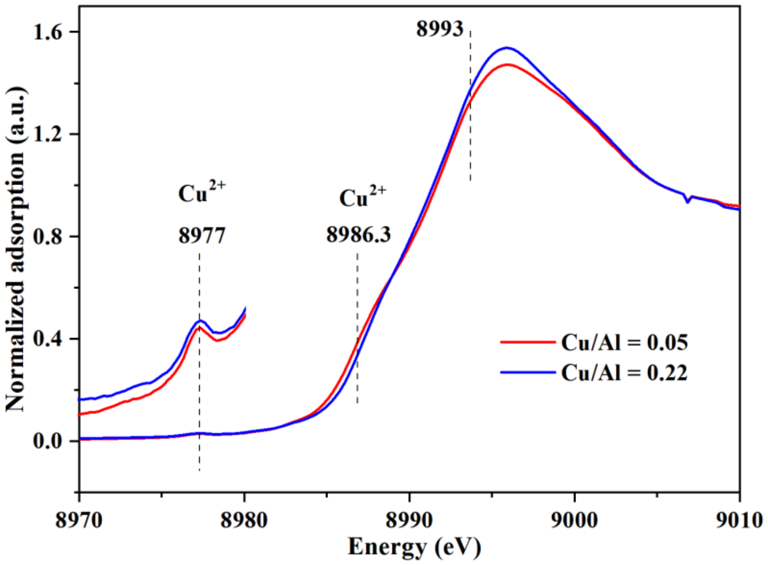

Fig. 7. XANES spectra of Cu/SSZ-13 catalysts with Cu/Al = 0.05 and 0.22.

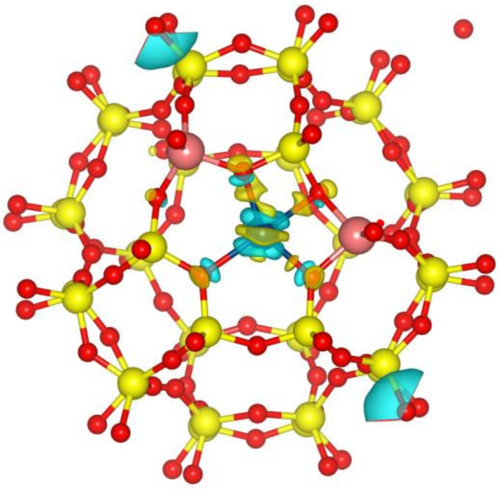

Fig. 8. Charge density differences (CDD, $\delta\rho = \rho_{total} - \rho_{Cu} - \rho_{the\ rest}$) of tetra-coordinated single-atom Cu(II) structure on 6MR of SSZ-13 (cyan stands for holes and yellow for electrons).

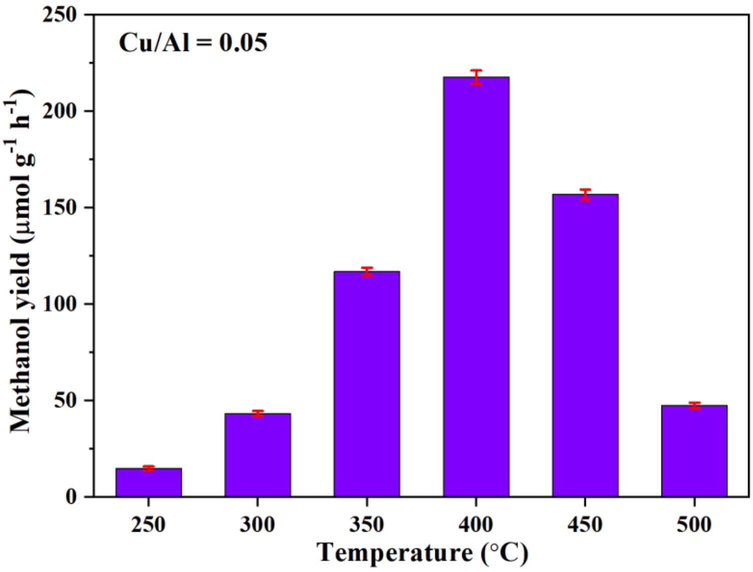

Fig. 9. Methanol yields (µmol g^{-1} h^{-1}) of Cu_1/SSZ-13 catalyst (Cu/Al = 0.05) in continuous methane-steam conversion to methanol with the increase of reaction temperature. Reaction conditions: 100 mg catalyst, total flow rate = 15 ml min^{-1}; 90% CH_4, 3.2% H_2O, He balance.

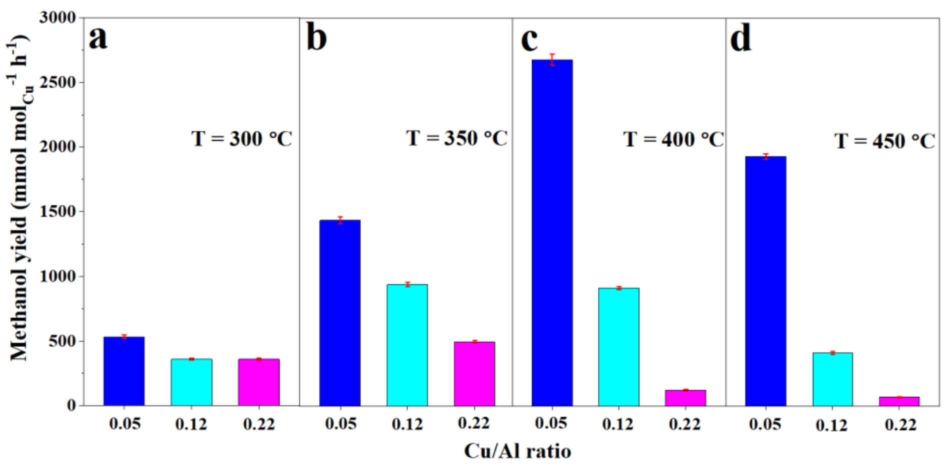

Fig. 10. Catalytic behaviors of Cu/SSZ-13 catalysts with different Cu/Al ratios in continuous methane-steam reaction towards methanol at the reaction temperatures of 300-450 °C (a-d). Reaction conditions: 100 mg catalyst, total flow rate = 15 ml min^{-1}; 90% CH_4, 3.2% H_2O, He balance.

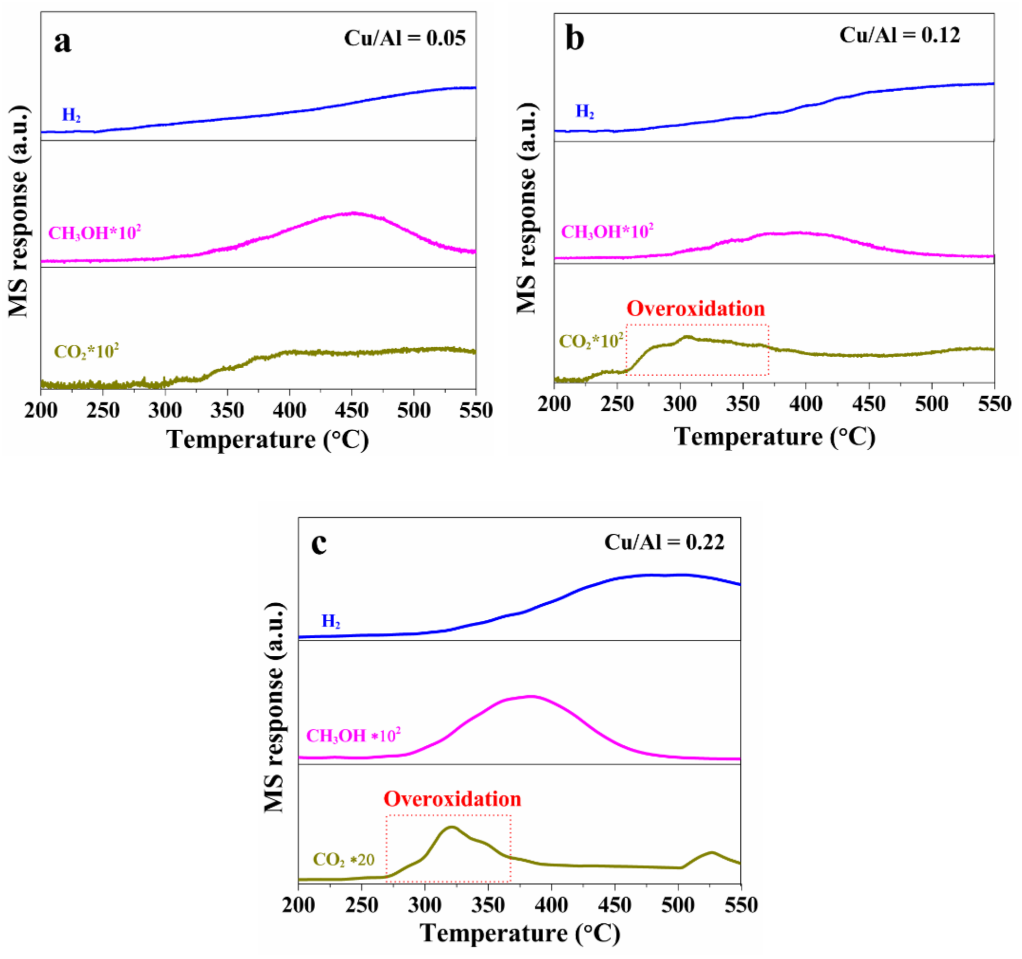

Fig. 11. Temperature-programmed surface reactions (TPSR) of methane oxidation on Cu/SSZ-13 with Cu/Al ratios = 0.05 (a), 0.12 (b) and 0.22 (c). Conditions: 100 mg catalyst, total flow rate of

15 ml min^{-1}; 90% CH_4, 3.2% H_2O, He balance; heating rate = 8 °C min^{-1}.

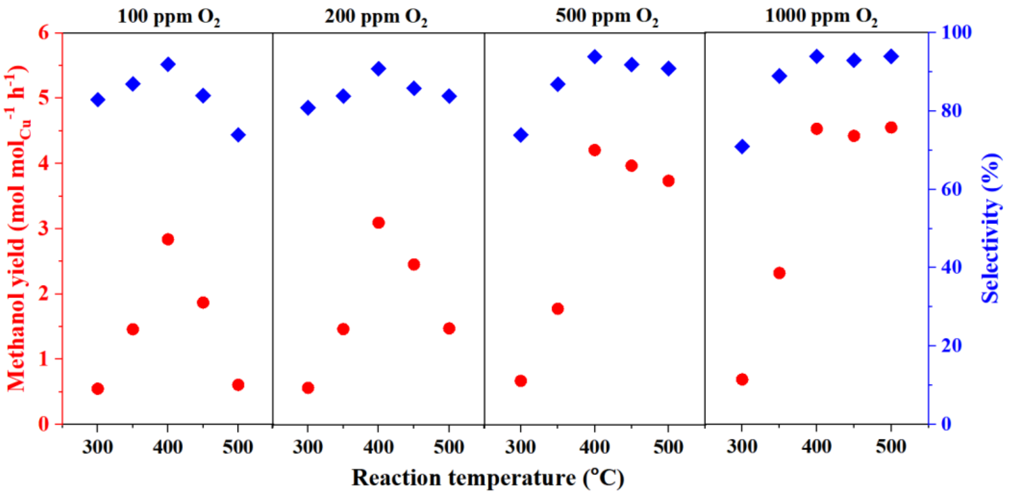

Fig. 12. Selective methane-steam conversion to methanol on Cu_1/SSZ-13 catalyst (Cu/Al = 0.05) under aerobic condition upon elevating reaction temperatures from 300 to 500 °C and increasing oxygen concentration from 100 to 1000 ppm. Red cycles and blue squares refer to methanol yield (mol mol_{Cu}^{-1} h^{-1}) and methanol selectivity (%), respectively.

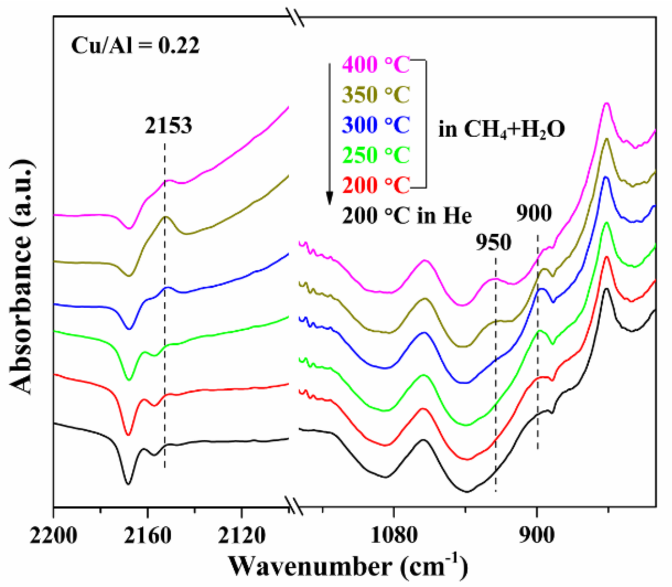

Fig. 13. *In situ* FTIR spectra of Cu/SSZ-13 catalyst with Cu/Al = 0.22 exposed in reaction gas mixture at different temperatures (200-400 °C). Conditions: ~30 mg catalyst, total flow rate of 15 ml min^{-1}; 90% CH_4, 3.2% H_2O, He balance. Prior to the measurement, the catalyst was pretreated in flowing He at 400 °C for 30 min, then the spectra were collected by subtraction of KBr background spectrum.

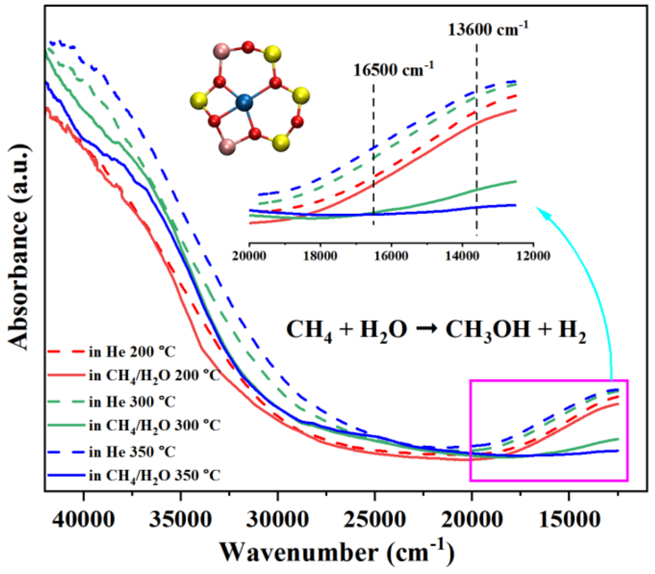

Fig. 14. *In situ* DR UV-Vis spectra of Cu/SSZ-13 sample with Cu/Al = 0.22 at different reaction temperatures (200-350 °C). Reaction conditions: mass of catalyst = 50-60 mg, the flow rate = 15 ml min^{-1}; 90% CH_4, 3.2% H_2O, He as balance gas; activation of the catalyst was conducted in the flowing He at 400 °C for 30 min, then UV-Vis spectra were collected in the flowing He and reaction gas at each reaction temperature, respectively, reaction time = 15 min; the UV-Vis spectra of $BaSO_4$ powders were used as the background spectra.

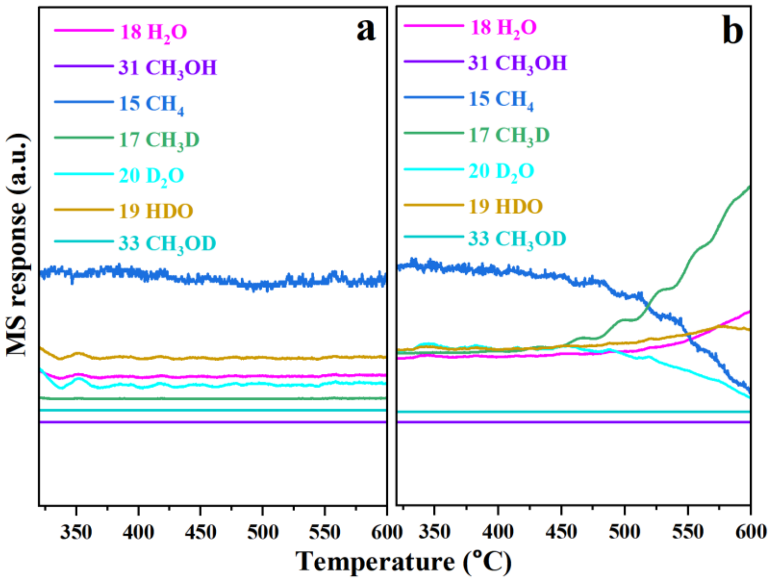

Fig. 15. The reference experiments of temperature-programmed surface reactions (TPSR) with isotope labeling CH_4-D_2O system, a: without any materials, only including the mixture of CH_4 + D_2O + He; b: with the zeolite of SSZ-13 without Cu site. Reaction conditions: 100 mg catalyst,

total flow rate = 15 ml min^{-1}; 96.8% methane, 3.2% water.

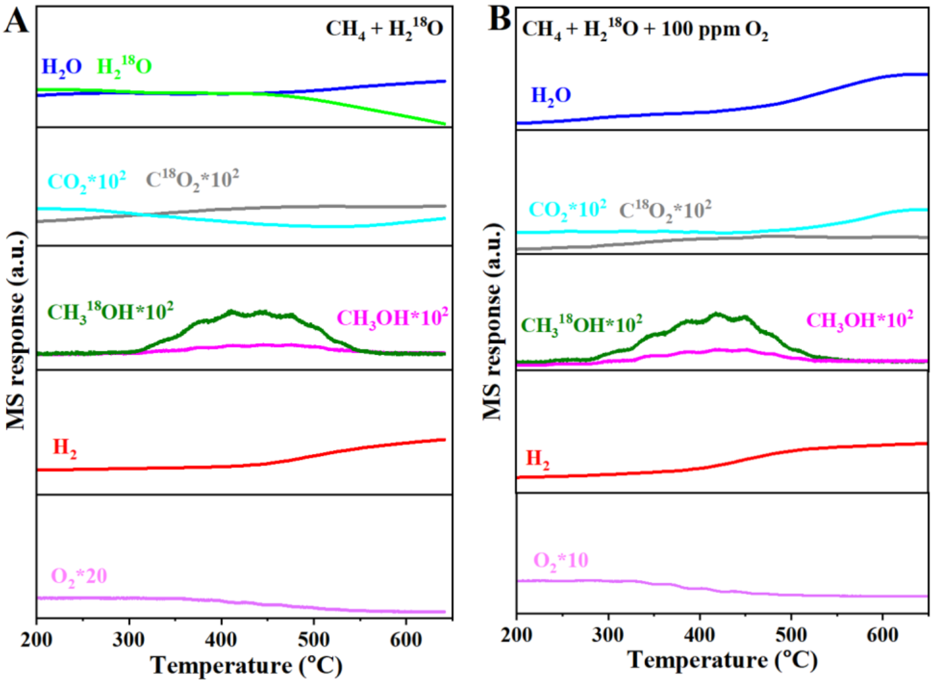

Fig. 16. Temperature-programmed surface reactions (TPSR) of continuous MTM on Cu_1/SSZ-13 catalyst with isotope labeling $CH_4 + H_2{}^{18}O$ (A) and $CH_4 + H_2{}^{18}O$ + 100 ppm O_2 (B) systems. Reaction conditions: 100 mg catalyst, total flow rate = 15 ml min^{-1}; 96.8% CH_4, 3.2% $H_2{}^{18}O$, 100 ppm O_2.

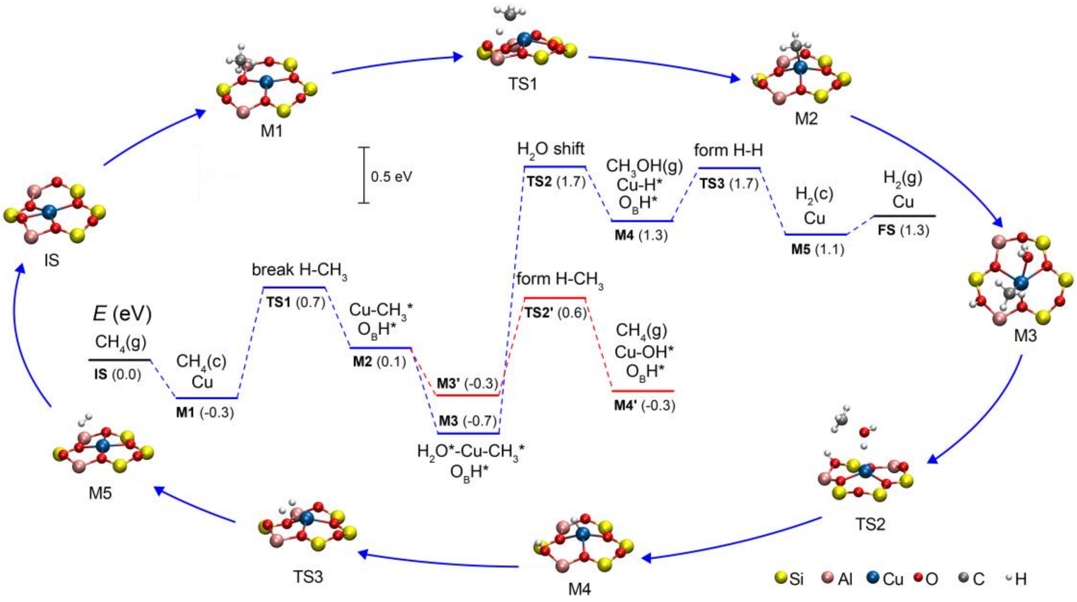

Fig. 17. The energy diagram of methane-to-methanol using water as oxidant on isolated Cu

single-atom site from 6MR of Cu_1/SSZ-13 catalyst (blue line), including the formation of monocopper Cu(II)-OH species (red line). The optimized ground-state structures of all intermediates (M1-M5) and transition states (TS1-TS3) were labeled around the pathway.

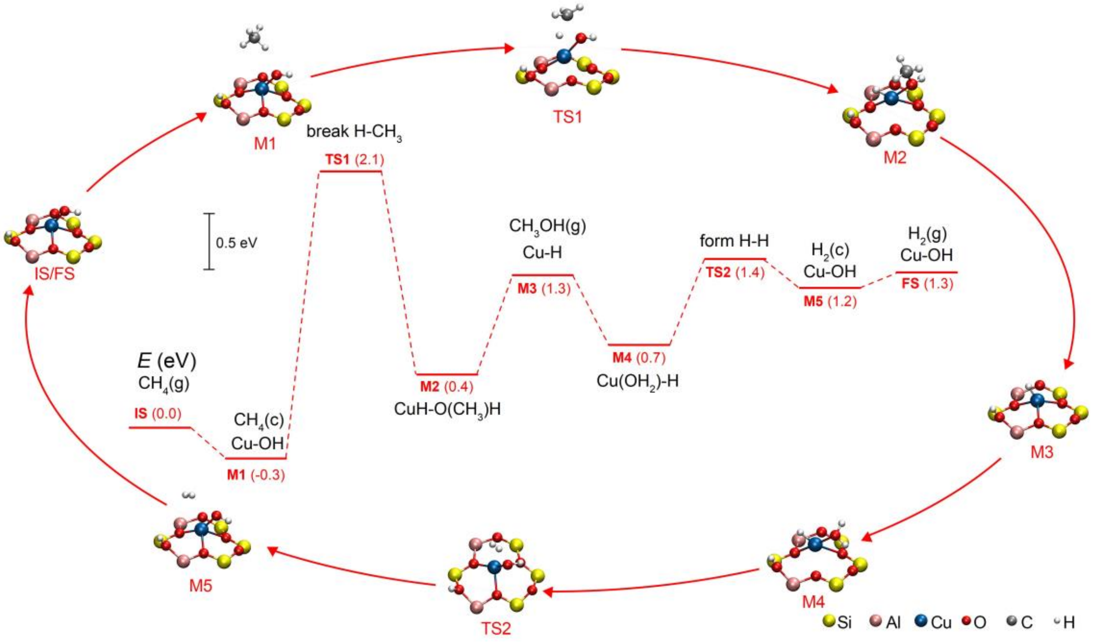

Fig. 18. The energy diagrams of the other MTM pathway on Cu-OH sites from 6MR voids, in which the methane C-H bond activation occurs on Cu atoms of Cu-OH sites. The optimized ground-state structures of all intermediates (M1-M5) and transition states (TS1-TS2) were labeled around the pathway.

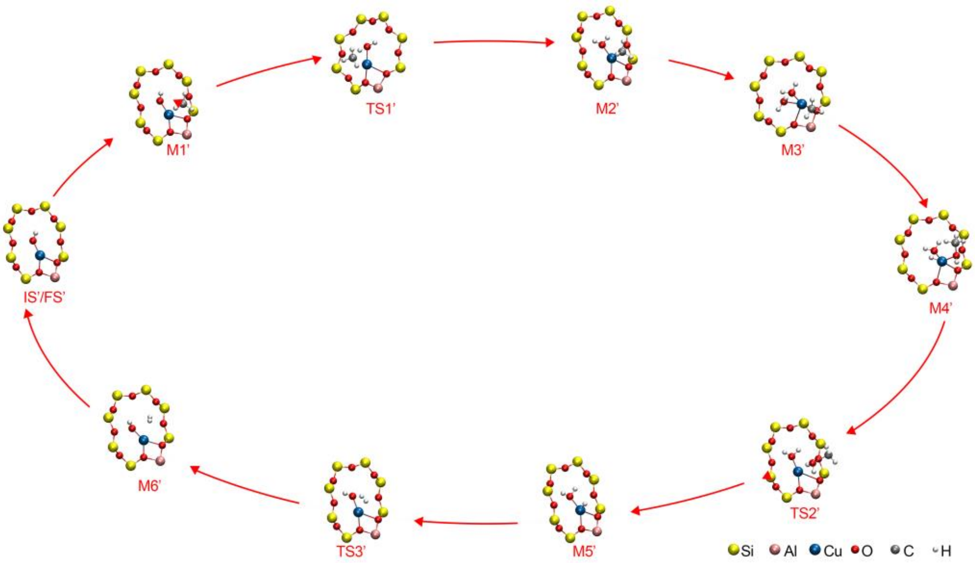

Fig. 19. The MTM reaction pathway on Cu-OH sites from 8-MR. The optimized ground-state

structures of all intermediates (M1'-M6') and transition states (TS1'-TS3') were labeled on the pathway.

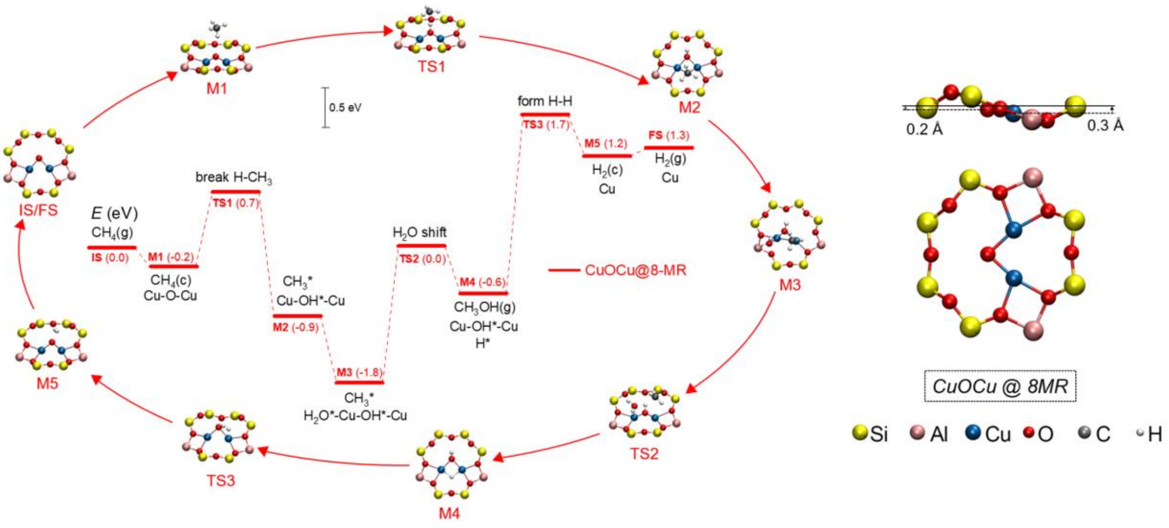

Fig. 20. The energy diagram of methane-to-methanol process using water as oxidant on a dimeric Cu cluster (Cu-O-Cu) residing in 8MR of Cu/SSZ-13 catalyst. The optimized structures of all intermediates (M1-M5) and transition states (TS1-TS3) were labeled around the pathway. The right showing the planar structure of Cu-O-Cu sites on 8MR.

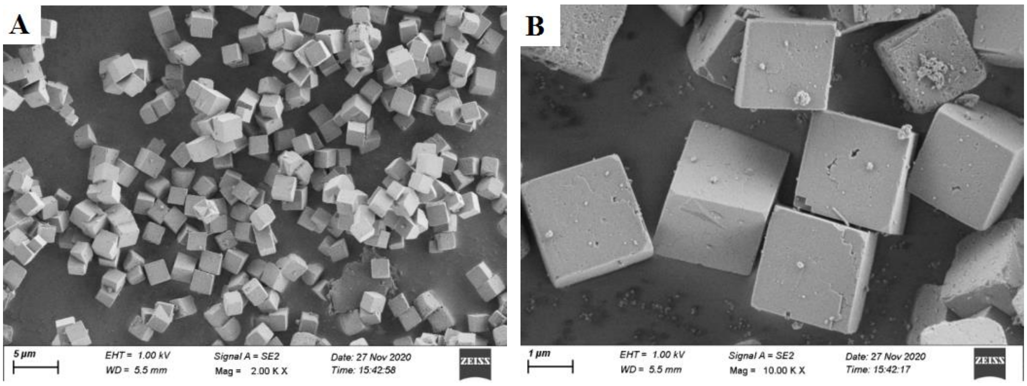

Fig. 21. The morphology of H-SSZ-13 zeolite with Si/Al molar ratio of 10.

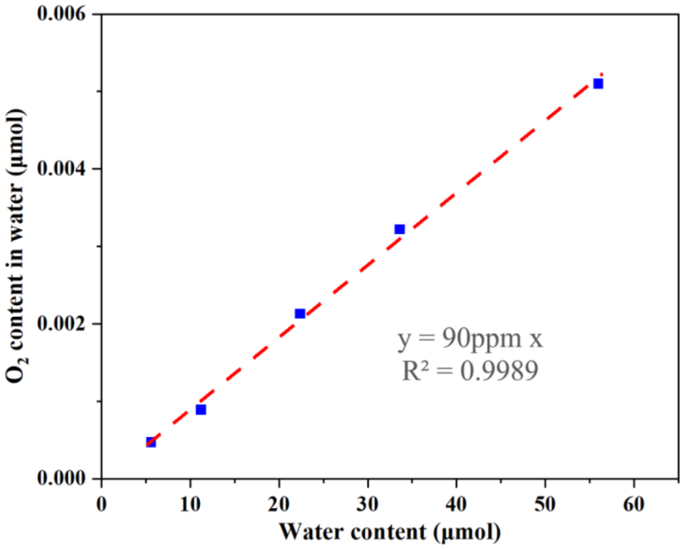

Fig. S22. The O_2 content in water. Prior to the measurement, the deionized water in an airtight saturator was heated up to 80 °C and kept at this temperature for 24 h in the flowing helium to remove the potential O_2 impurity in water.

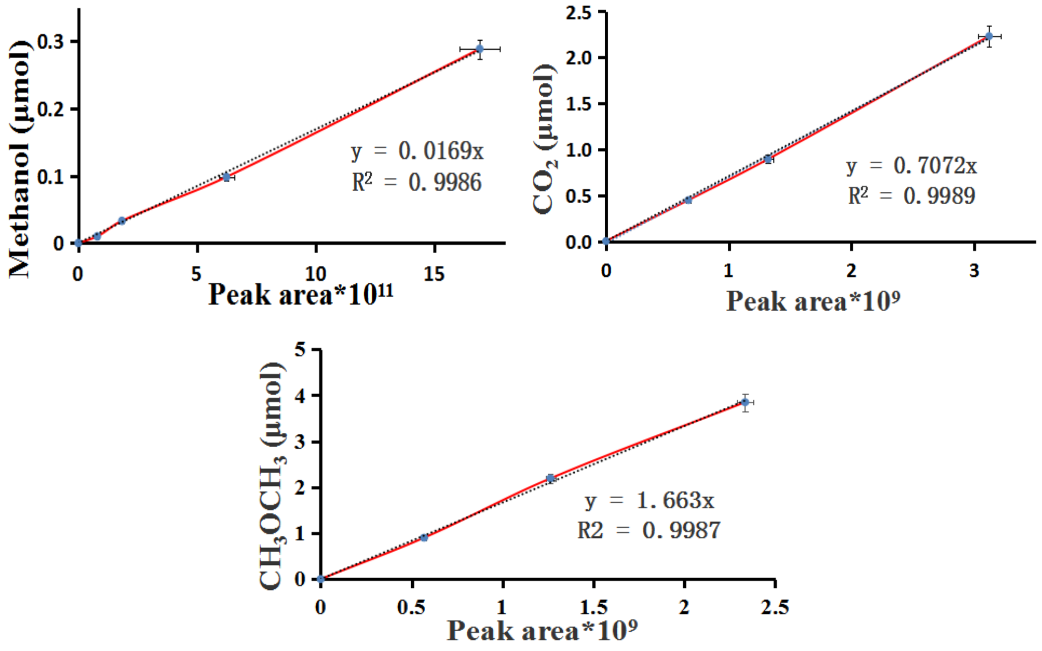

Fig. 23. The calibration curves of CH_3OH (m/z = 31), CO_2 (m/z = 44) and $(CH_3)_2O$ (m/z = 45) obtained by an external standard method.

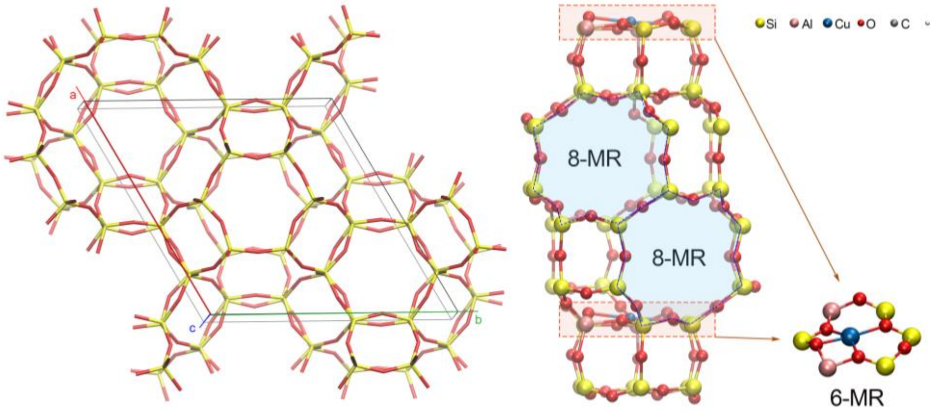

Fig. 24. The model for Cu_1/SSZ-13 zeolite with 6MR-hosted bare copper(II) single atom was built as the initial state of active site.

Tables

Table 1. The Cu loadings and textual property of Cu/SSZ-13 catalysts.

Cu-SSZ-13	Sample 1	Sample 2	Sample 3
Cu loading (wt.%)[a]	0.52	1.2	2.2
Cu/Al ratio[a]	0.05	0.12	0.22
Specific surface area (m^2/g)[b]	535	527	521
Average pore diameter (nm)[c]	4.2	3.9	3.4

[a]obtained by WDXRF.

[b]collected via Brunauer-Emmett-Teller (BET) method.

[c]obtained by Barret-Joyner-Halenda (BJH) method.

Table 2. Fitting parameters of the curves fitted k^2-weighted EXAFS analysis of the Cu-exchanged SSZ-13 samples with Cu/Al = 0.05 and 0.22.

Sample	Shell	N	R(Å)	ΔE_0 (eV)	S_0^2	R-factor
Cu/Al = 0.05	Cu-O	4.02	1.95	3.636	0.98	0.0058
Cu/Al = 0.22	Cu-O	3.49	1.96	3.385	0.98	0.0099

N: coordination number; R: distance between absorber and backscatered atoms; ΔE_0: inner potential correction; R-factor: closeness of the fit, if < 0.02, consistent with broadly correct models.

Table S3. Comparison of methanol yields and selectivities on Cu-zeolites in methane to methanol conversion.

Catalyst[a]	Cu/Al	Conditions	Productivity[b]	Sel. (%)	STY[c]	Ref.
Cu/MFI	0.31	450 °C in O_2; 175 °C in CH_4; RT in H_2O/CH_3CN	~27	/	/	1
Cu/MOR	0.43	450 °C in O_2; 175 °C in CH_4; RT in H_2O/CH_3CN	~19	/	/	1
Cu/MOR	0.38	450 °C in O_2; 200 °C in CH_4; RT in H_2O	19	98	/	2
Cu/SSZ-13	0.35	450 °C in O_2; 200 °C in CH_4; 200 °C in H_2O	~60	95	/	3
Cu/SSZ-16	0.34	450 °C in O_2; 200 °C in CH_4; 200 °C in H_2O	~50	95	/	3
Cu/SSZ-39	0.26	450 °C in O_2; 200 °C in CH_4; 200 °C in H_2O	~90	95	/	3
Cu/MOR	/	450 °C in He; 200 °C in CH_4; 135 °C in H_2O	310	80	/	4
Cu/CHA	/	210 °C in 98.1% CH_4 + 3.2% H_2O + 25 ppm O_2	/	/	6.1	5
Cu/MFI	0.31	210 °C in 98.1% CH_4 + 3.2% H_2O + 25 ppm O_2	238 (108 h)	70.6	2.2	5
Cu/MOR	0.28	450 °C in He; 200 °C in CH_4; 135 °C in H_2O	~230	~75	/	6
Cu/MOR	/	450 °C in He; 200 °C in CH_4 (36 bar); 200 °C in H_2O	141	/	/	7
Cu/SSZ-13	0.4	260 °C in 30% CH_4 + 30% N_2O + 3% H_2O	/	27	38	8
Cu/SSZ-13	0.4	300 °C in 30% CH_4 + 30% N_2O + 3% H_2O	/	2.3	110	8
Cu/SSZ-13	0.4	300 °C in 30% CH_4 + 30% N_2O + 20% H_2O	/	12.5	98	8
Cu/SSZ-13	0.4	300 °C in 30% CH_4 + 30% O_2 + 3% H_2O	/	2	18	8
Cu/MOR	0.38	400 °C in He; 200 °C in CH_4 (7 bar); 200 °C in H_2O (7 bar)	204	97	/	9

Cu/MOR	0.4	450 °C in O_2; 150 °C in CH_4; 135 °C in H_2O	215	/	/	10
Cu/MOR	0.4	600 °C in N_2O; 150 °C in CH_4; 135 °C in H_2O	311	/	/	10
Cu/CHA	0.5	500 °C in O_2; 200 °C in CH_4; 200 °C in H_2O	200 (6 h)	90	/	11
Cu/SSZ-13	0.47	450 °C in O_2; 50 °C in CH_4; 200 °C in H_2O	50	>95	/	12
Cu/SSZ-39	0.48	450 °C in O_2; 50 °C in CH_4; 200 °C in H_2O	35	>95	/	12
Cu/MOR	0.60	400 °C in O_2; 200 °C in CH_4 (7 bar); 200 °C in H_2O (7 bar)	316	98	/	13
Cu/MOR	0.38	400 °C in O_2; 200 °C in CH_4 (7 bar); 200 °C in H_2O (7 bar)	142	87	/	13
Cu/MOR	0.38	400 °C in He; 200 °C in CH_4 (7 bar); 200 °C in H_2O (7 bar)	204	97	/	13
Cu/MOR	0.18	500 °C in N_2O; 150 °C in CH_4; 135 °C in H_2O	470	89	/	14
Cu/MOR	0.28	450 °C in O_2; 200 °C in CH_4 (35 bar); 200 °C in H_2O	420 (20 h)	90	/	14
Cu/MOR	0.28	450 °C in O_2; 200 °C in CH_4 (1 bar); 200 °C in H_2O	330 (70 h)	80	/	14
Cu/MOR	0.28	450 °C in O_2; 200 °C in CH_4 (35 bar); 200 °C in H_2O	390 (20 h)	90	/	15
Cu/ZSM-5	0.26	450 °C in O_2; 200 °C in CH_4 (35 bar); 200 °C in H_2O	270 (20 h)	83	/	15
Cu/MAZ B	/	450 °C in O_2; 200 °C in CH_4; 200 °C in H_2O	220	/	/	16
Cu/SSZ-13	0.22	P_{CH4} = 18 kPa, P_{O2} = 0.09 kPa, P_{H2O} = 3.14 kPa, bal He	/	95	~37.6	17
Cu/SSZ-13	/	265 °C in CH_4 + O_2 + H_2O	/	53	26	17
Cu/SSZ-13	0.33	225 °C in 49.5% CH_4 + 2.3% H_2O	/	/	10.9	18
Cu/MAZ	/	450 °C in O_2; 200 °C in CH_4 (6 bar);	270	/	/	19

		extraction in H_2O				
Cu/MOR	0.06	500 °C in O_2; 200 °C in CH_4; 200 °C in H_2O	300	84	/	20
Cu/CHA	0.23	300 °C in 98% CH_4 + 2% H_2O + 400 ppm O_2	/	91	543	21
Cu/CHA	0.23	400 °C in 98% CH_4 + 2% H_2O + 400 ppm O_2	/	~58	~585	21
Cu/MFI	0.27	300 °C in 98% CH_4 + 2% H_2O + 200 ppm O_2	/	~68	~150	21
Cu/MOR	0.27	300 °C in 98% CH_4 + 2% H_2O + 200 ppm O_2	/	~30	~25	21
Cu/MOR	0.145	350 °C in 33% CH_4 + 67% H_2O	/	~100	~16.5	22
Cu/MOR	0.145	400 °C in 33% CH_4 + 67% H_2O	/	~100	~18	22
Cu/MOR	0.185	350 °C in 33% CH_4 + 67% H_2O	/	~100	~22	22
Cu/MOR	0.185	400 °C in 33% CH_4 + 67% H_2O	/	~100	~17	22
Cu/CHA	0.8	200 °C in 0.2 MPa O_2 + 0.5 MPa CO + 2.0 MPa CH_4 + 5 ml H_2O	/	/	80	23
Cu/SSZ-39	0.22	325 °C in 40.5% CH_4 + 15.2% N_2O + 4.0% H_2O	/	34	~1024	24
Cu/SSZ-13	0.10	300 °C in 40.5% CH_4 + 10.1% N_2O + 2.6% H_2O	/	59	157	24
Cu/MOR	~0.23	300 °C CH_4 + O_2 + H_2O	/	/	308.3	25
Cu_1/SSZ-13	0.05	400 °C in 90% CH_4 + 3.2% H_2O	~53000 (20 h)	**~93**	**2678**	This work

[a]Obtained by metal ion-exchanged method.

[b]Methanol productivity, mmol mol_{Cu}^{-1}.

[c]Methanol space-time yield (STY), mmol mol_{Cu}^{-1} h^{-1}.

Table 4. The m/z values for the chemicals used in this study and their relative intensities.

Chemicals	m/z	Chemicals	m/z
H_2	2 (100%), 1 (1%)	CH_3OCH_3	45 (100%), 46 (61%), 29 (39%)
D_2	4 (100%), 2 (1%)	CH_3OH	31 (100%), 32 (74%), 29 (43%)
HD	3 (100%), 2 (1%), 1 (1%)	CH_3OD	32 (100%), 33 (74%), 29 (43%)
H_2O	18 (100%), 17 (21%), 16 (1%)	CH_2DOD	32 (100%), 34 (74%), 31 (40%)
$H_2{}^{18}O$	20 (100%), 19 (21%), 18 (%)	CHD_2OD	33 (100%), 35 (74%), 30 (40%)
D_2O	20 (100%), 18 (21%), 16 (1%)	CD_3OD	34 (100%), 36 (71%), 30 (50%)
HDO	19 (100%), 18 (21%), 16 (1%)	$CH_3{}^{18}OH$	33 (100%), 34 (74%), 31 (43%)
CH_4	16 (100%), 15 (85%), 14 (16%)	CO_2	44 (100%), 28 (50%), 16 (50%)
CH_3D	17 (100%), 16 (76%)	$C^{18}O_2$	48 (100%), 30 (50%), 18 (50%)
$CH_3{}^{18}OCH_3$	47 (100%), 48 (61%), 31 (39%)	O_2	32 (100%), 16 (22%)

Indicative m/z values shown in red; relative intensities shown in parentheses.

Analysis and discussion

FTIR simulation by DFT calculation

In the present work, we also present the FTIR spectrum simulations in T-O-T bond vibration region of Cu-CHA zeolite via DFT calculations using the PHONOPY code (version: 2.12.0)[26]. As shown in Fig. 2, the tetra-coordinated Cu(II) single-atom site on 6MR has a strong IR vibration at ~900 cm^{-1}, while the monocopper Cu-OH site on 8MR exhibits the strong IR vibration at ~925 and ~993 cm^{-1}. The former shows a lower vibration wavenumber than the latter, because the interaction between the Cu single atom and the 6MR framework is stronger. The DFT-simulated FTIR vibration bands corresponding to the bare Cu single-atom hosted on 6MR void and the Cu-OH single site located on 8MR void are basically accordant with the experimental results. However, the band intensity of Cu-OH single site hosted on 6MR is very weak. Besides, the DFT simulation also presents that the strong FTIR vibration band at ~950 cm^{-1} belongs to the Cu-CH_3 species formed from methane C-H bond activation over the Cu single-atom site confined in the 6MR void.

H_2 temperature-programmed reduction (H_2-TPR)

H_2 temperature-programmed reduction (H_2-TPR) has been widely used to differentiate two different Cu species hosted in SSZ-13 zeolite[27-31]. It was reported by Gao et al. that the reduction of all the Cu^{2+} species in Cu/SSZ-13 catalyst occurred at lower than 600 °C[32, 33]. Fig. 4 shows the H_2-TPR results of Cu ion-exchanged SSZ-13 zeolites with different Cu/Al ratios (0.05-0.22), which present different H_2-TPR profiles with the reduction temperatures lower than 600 °C. The catalyst with Cu/Al = 0.05 (hereinafter denoted as Cu_1/SSZ-13) exhibits only one reduction peak at ~390 °C. The other reduction peak at ~250 °C appears when the Cu/Al ratio increases from 0.05 to 0.22. Previous works revealed that the different reduction temperatures of Cu(II) species in TPR profiles are related to the stability of Cu^{2+} coordinated to

different positions of zeolites[27, 32, 33]. Generally, the Cu^{2+} cations with coordination to four lattice oxygen atoms neighboring two framework Al sites on 6MRs are more stable than the Cu-OH (or $[CuOH]^+$) species coordinated to oxygen atoms near one Al site on 8MR in CHA cages. Thus, the reduction of the former is required a higher temperature compared to the other Cu species[27, 28, 32-35]. Based on this point, the reduction peak at ~390 °C is ascribed to the bare tetra-coordinated Cu(II) species on 6MR and that at ~250 °C is assigned to the Cu-OH species on 8MR.

DR UV-Vis spectroscopy

Fig. 5 presents DR UV-Vis spectra of Cu ion-exchanged zeolites with different Cu/Al ratios (0.05-0.22). Based on the previous report by Ipek et al.,[12] the trans-μ-1,2-peroxo dicopper(II) ($[Cu_2O_2]^{2+}$) and mono-(μ-oxo) dicopper(II) ($[Cu_2O]^{2+}$) species formed in Cu/SSZ-13 can be identified by DR UV-Vis spectroscopy in the energy range of 22200-35000 cm^{-1}. The UV-Vis bands in the energy range cannot be found in the UV-Vis spectrum of Cu_1/SSZ-13 with Cu/Al = 0.05, only the band at ~13600 cm^{-1} is observed, which is assigned to the bare Cu(II) single-atom confined on 6MR. While the formation of Cu_xO_y clusters is confirmed at higher Cu loadings (Cu/Al = 0.12 and 0.22). Such cluster motifs are generally hosted in larger 8MR voids.

X-ray absorption spectroscopy (XAS) results

EXAFS fittings of the XAS data were conducted to investigate the 1st shell coordination structures of the Cu atoms in Cu/SSZ-13 catalysts (Fig. 6). EXAFS analysis reveals that the average coordination number of the Cu atoms from Cu_1/SSZ-13 (Cu/Al = 0.05) is 4.02. Such a tetra-coordinated structure of Cu atom is impossible to form in larger 8MR void of SSZ-13 zeolite, since it is unstable. On the contrary, it is more stable in small 6MR void with tetra-coordination to oxygen atoms neighboring two Al sites. In fact, the Cu species on 8MR, including Cu-oxo clusters (e.g., $[Cu_2O]^{2+}$ and $[Cu_2O_2]^{2+}$) and monocopper $[CuOH]^+$ sites, appeared in the form

of tri-coordinated structure when the Cu/Al ratio increased to 0.22. The average coordination number of the Cu atoms at Cu/Al = 0.22 is revealed to be 3.49 (Table 2), which is due to the co-existence of tri-coordinated structure in large 8MR void and tetra-coordinated structure in small 6MR void of CHA zeolite. In Fig. 7, XANES spectra of Cu/SSZ-13 show a preedge band at 8977 eV and absorption edge energy of 8993 eV with a shoulder band at 8986.3 eV, which indicates the presence of a dominant oxidation state of Cu^{2+} [9].

Charge density difference (CDD) analysis

Fig. 8 shows the charge density differences (CDD, $\delta\rho = \rho_{total} - \rho_{Cu} - \rho_{the\ rest}$) of tetra-coordinated structure of Cu(II) single-atom site confined within 6MR based on DFT calculations. The result indicates the electron transfer between Cu atom and four oxygen atoms from 6MR framework, thus the formation of Cu-O chemical bonds is very possible.

Temperature-programmed surface reactions (TPSR)

Fig. 11 shows the temperature-programmed surface reactions (TPSR) of methane-steam conversion to methanol on Cu/SSZ-13 catalysts. Methane oxidation started at 300-350 °C for Cu_1/SSZ-13 catalyst (Cu/Al = 0.05) with only bare Cu single-atom site on 6MR and at 250-300 °C for the other Cu/SSZ-13 catalysts with higher Cu/Al ratios (0.12 and 0.22), producing methanol and dihydrogen as the desired products and CO_2 as the main byproduct using water as the oxidant. It is worth noting that an apparent CO_2 peak (red square) is observed at the initial stage of methane oxidation for Cu/Al = 0.12 and 0.22 (Figs. 11b and c) and the latter presents higher CO_2 production. This illustrates that the over-oxidation of methane occurred on the catalysts with higher Cu loadings (Cu/Al = 0.12 and 0.22), in which the Cu_xO_y clusters and Cu-OH species confined in 8MR voids appeared as shown in the previous characterizations (Figs. 4-5). However, this phenomenon cannot be observed on

Cu_1/SSZ-13 catalyst (Fig. 11a), revealing a very weak ability of the 6MR-hosted Cu single-atom site for the over-oxidation of methane into CO_2. The previous UV-Vis spectra of Cu/SSZ-13 (Fig. 5) confirmed an increased concentration of Cu_xO_y clusters confined in 8MR as the Cu/Al ratio increased from 0.05 to 0.22, which would induce the over-oxidation of methane due to a high oxidation ability of active oxygen from Cu_xO_y clusters. At higher reaction temperatures, the production of CO_2 and H_2 mainly resulted from the methanol reforming ($CH_3OH + H_2O \rightarrow CO_2 + 3H_2$).[21]

Activity tests for MTM under aerobic conditions

The activity tests in the case of aerobic conditions were also conducted and the results are shown in Fig. 12. It is revealed that the methanol productivity has an apparent increase after introducing O_2 at higher temperatures (> 350 °C), but there is no a visible increase in lower reaction temperatures (< 350 °C). Furthermore, it was found that increasing the O_2 concentration to 500-1000 ppm resulted in a much higher yield of methanol at 400-500 °C (~4-4.5 mol mol_{Cu}^{-1} h^{-1}), presenting a higher methanol selectivity (~93%). However, the small amount of oxygen in the feed (< 200 ppm) did not lead to a larger increase in methanol yields at the same temperatures. Such a high methanol yield under 500-1000 ppm O_2 should be attributed to the role of oxygen in selective-oxidation of methane to methanol and/or the cooperative effect of O_2 and H_2O.[21] Interestingly, the single Cu active sites confined on the 6MR as a small void present a much higher methanol yield at higher temperatures (> 400 °C) under aerobic conditions, which is different with the Cu-OH and Cu-oxo cluster active sites hosted on 8MR, as reported in the previous work, increasing the reaction temperature resulted in a lower methanol yield and much higher CO_2 production.[21]

In situ FTIR spectroscopy

Fig. 13 shows *in situ* FTIR spectra of Cu/SSZ-13 catalyst with Cu/Al = 0.22 in continuous methane-steam conversion to methanol at different reaction temperatures

(200-400 °C). It was found that increasing the reaction temperature from 300 to 400 °C led to a decrease in the band intensity at ~900 cm^{-1} and an increase in the band intensity at ~950 cm^{-1}, accompanied with the appearance of the band at 2153 cm^{-1} as the featured band of Cu^{+}-CO species. This indicates the consumption of the tetra-coordinated Cu single-atom sites confined within 6MR and the formation of Cu-CH_3 species from methane C-H bond activation (the IR band at ~950 cm^{-1} belonging to Cu-CH_3 is based on the DFT calculations (Fig. 2)).

In situ FTIR spectra of Cu_1/SSZ-13 catalyst were recorded by exposure in the reaction gas (90% CH_4 + 3.2% H_2O) at 400 °C using the catalyst itself as the background (see Fig. 3d in main text). We found that a negative band at ~900 cm^{-1} and a positive band at ~950 cm^{-1} appeared after introducing the reaction gas, illustrating the consumption of tetra-coordinated Cu single-atom sites and the formation of Cu-CH_3 species on 6MR during methane-steam conversion. Meanwhile, the Cu^{+}-CO species and Brønsted acid sites were also detected at ~2153 and ~3585 cm^{-1}, respectively. This reveals that a Cu^{2+}-Cu^{+} cycle occurred and the amount of B acid sites increased during MTM. The IR bands at 1300, 1344, 2920, 3017 and 3090 cm^{-1} are assigned to the characteristic bands of methane. When the catalyst was exposed in He flow after MTM, the bands that are assigned to B acid sites (~3585 cm^{-1}) and Cu-OH species (3655 cm^{-1}) were detected[21, 35]. Note the adsorbed methane on the catalyst was still detected in He flow, thus the band at ~950 cm^{-1} is assigned to Cu-CH_3 species.

In situ UV-Vis spectroscopy

To further confirm the bare Cu single-atoms on 6MRs are active sites for methane-steam conversion to methanol, the *in situ* DR UV-Vis spectroscopy of Cu/SSZ-13 catalyst (Cu/Al = 0.22) were measured at 200, 300 and 350 °C, respectively. Fig. 14 presents the UV-Vis spectra of the catalyst in methane-steam conversion at the elevated temperatures. The characteristic bands of the 6MR-hosted bare Cu site are identified at 13600 and 16500 cm^{-1}. At reaction temperature of 200 °C, almost no changes in the intensities of both the bands are found, only a

decrease in absorption intensity at 30000-35000 cm^{-1} is observed, which is assigned to the Cu-oxo cluster active species. The result illustrates that the bare Cu(II) single-atom is not active at 200 °C, while the Cu_xO_y clusters (e.g., $[CuOCu]^{2+}$) are main active species for MTM. After raising the temperature up to 300 and 350 °C, it can be observed in Fig. 14 that the intensities of the absorption bands at 13600 and 16500 cm^{-1} apparently decrease. This demonstrates that the 6MR-confined Cu single sites are active only at higher temperatures, because their tetra-coordination structure is more stable compared with the active sites on 8MRs.

Isotope labeling experiments

The reference experiment using the same SSZ-13 zeolite without Cu was carried out in this study, the result is shown in Fig. 15. It was found that the H from CH_4 molecule was exchanged with the H from H_2O, which is similar with the result shown in Fig. 4 in the main text. However, such a result could not be found in a reference experiment without any materials (Fig. 15a). This illustrates that the exchange of H atoms between CH_4 and H_2O occurred on the surface of SSZ-13 zeolite, which is attributed to the coordination of CH_4/H_2O to Brønsted acid sites. Besides, it is worth noting that the H-D exchanges occurred at different temperatures comparing the reference experiment (Fig. 15b) with the results in Fig. 4 shown in the main text. It was found that the H atoms from CH_4 were apparently exchanged at > 450 °C in the reference experiment without Cu (SSZ-13), while such a chemical process started to occur at lower temperatures (~350 °C) in the presence of Cu (Cu_1/SSZ-13). This indicates that the H exchange also took place in the Cu sites.

The labeling experiments with $H_2{}^{18}O$ were also carried out in methane-steam reactions, the results are shown in Fig. 16. In Fig. 16A, it was found that the MS signals of O_2 decrease at > 300 °C, this indicates that a small amount of ambient O_2 participated in the chemical reactions. However, we did not find the apparent MS signals of CH_3OH and CO_2, except for the visible observation of $CH_3{}^{18}OH$ and $C^{18}O_2$ signals, together with the increase of H_2O and the decrease of $H_2{}^{18}O$. The similar

results were also found in the reaction system of CH_4 + $H_2{}^{18}O$ + 100 ppm O_2 (Fig. 16B). These results illustrate that that water acted as the dominant oxidant in methane conversion.

In our opinion, the O_2 participated the reaction, but the oxygen source of methanol is only water, that is, the O_2 would not directly participate MTM reactions. In the labeling experiments, we found that $H_2{}^{18}O$ was consumed together with the production of H_2O, it is very possible that the H_2 produced from the reaction of CH_4 and $H_2{}^{18}O$ reacted with O_2 to form H_2O: $CH_4 + H_2{}^{18}O \rightarrow CH_3{}^{18}OH + H_2$, $H_2 + O_2 \rightarrow H_2O$ (overall reaction: $2CH_4 + 2H_2{}^{18}O + O_2 \rightarrow 2CH_3{}^{18}OH + 2H_2O$). Then the H_2O from H_2 oxidation participated the MTM reaction, thus a small amount of CH_3OH was found. In fact, the fast consumption of H_2 as the MTM product can enhance the production of CH_3OH.

DFT calculation analysis

Fig. 17 shows the energy diagram and reaction pathway of MTM using water as the oxidant on Cu single-atom site from 6MR, which was set as the initial active site for methane conversion. The activation of methane resulted in the evolution of tetra-coordinated copper into a Cu-CH_3* structure (**M2**), accompanied by the formation of Brønsted acid site (O_BH) on 6MR with energy barrier of 0.7 eV. The subsequent adsorption of water produced a Cu complex (H_2O*-Cu-CH_3*, **M3**), which then desorbed a methanol molecule via the transition state (**TS2**) at a high energy barrier of 1.7 eV and converted to Cu-H* species (**M4**). Finally, the combination of the H atoms from Cu-H* and O_BH* produced H_2, with the regeneration of tetra-coordinated single-atom Cu active site (**M5**). Besides, it is worth noting that a methane molecule re-formed via the methyl on Cu-CH_3* abstracting a H from water dissociation, accompanied with the formation of monocopper Cu-OH species as the potential active sites (red line, **M3′→M4′**). This pathway is shown to require a lower energy barrier of 0.6 eV (**TS2′**) in comparison with **TS2** on the blue line. Thus, it is rational to propose that the pathway with red line is main reaction path, although we cannot rule out the possibility of direct MTM conversion pathway (the blue line) on

the bare Cu single-atom site confined within the 6MR void.

Fig. 18 shows the other reaction pathways of MTM on Cu-OH sites as the active species from 6MR. A C-H bond in the methane was firstly activated on the Cu atom of Cu-OH site via a transition state (**TS1**) with a very high energy barrier of 2.1 eV to form CuH-O(CH_3)H species (**M2**). Then the methanol was desorbed from the copper active site at energy barrier of 1.3 eV, accompanied by the formation of Cu-H species (**M3**). Subsequently, the Cu-H site adsorbed a water molecule to form Cu(OH_2)-H species (**M4**) in the presence of excessive water. Finally, the Cu-OH active site was regenerated via the H_2 formation (**M5**). Such reaction pathway presents much higher activation energy (2.1 eV) of methane C-H bonds than the activation pathway occurred on oxygen atoms from Cu-OH sites (0.6 eV).

References:

1. Groothaert, M. H. et al. Selective oxidation of methane by the bis (μ-oxo) dicopper core stabilized on ZSM-5 and mordenite zeolites. *J. Am. Chem. Soc.* **127**, 1394-1395 (2005).
2. Alayon E. M. et al. Catalytic conversion of methane to methanol over Cu-mordenite. *Chem. Commun. (Camb)* **48**, 404-406 (2012).
3. Wulfers M. J. et al. Conversion of methane to methanol on copper-containing small-pore zeolites and zeotypes. *Chem. Commun. (Camb)* **51**, 4447-4450 (2015).
4. Grundner S. et al. Single-site trinuclear copper oxygen clusters in mordenite for selective conversion of methane to methanol. *Nat. Commun.* **6**, 7546 (2015).
5. Narsimhan K. et al. Catalytic Oxidation of Methane into Methanol over Copper-Exchanged Zeolites with Oxygen at Low Temperature. *ACS Cent. Sci.* **2**, 424-429 (2016).
6. Grundner S. et al. Synthesis of single-site copper catalysts for methane partial oxidation. *Chem. Commun. (Camb)* **52**, 2553-2556 (2016).
7. Tomkins P. et al. Isothermal Cyclic Conversion of Methane into Methanol over Copper-Exchanged Zeolite at Low Temperature. *Angew. Chem. Int. Ed. Engl.* **55**, 5467-5471 (2016).
8. Ipek B. et al. Catalytic conversion of methane to methanol on Cu-SSZ-13 using N_2O as oxidant. *Chem. Commun. (Camb)* **52**, 13401-13404 (2016).
9. Sushkevich, V. L. et al. Selective anaerobic oxidation of methane enables direct synthesis of methanol. Science **356**, 523-527 (2017).
10. Kim Y. et al. Distinct activation of Cu-MOR for direct oxidation of methane to methanol. *Chem. Commun. (Camb)* **53**, 4116-4119 (2017).
11. Pappas D. K. et al. Methane to Methanol: Structure-Activity Relationships for Cu-CHA. *J. Am. Chem. Soc.* **139**, 14961-14975 (2017).
12. Ipek B. et al. Formation of $[Cu_2O_2]^{2+}$ and $[Cu_2O]^{2+}$ toward C–H bond activation in Cu-SSZ-13

and Cu-SSZ-39. *ACS Catal.* **7**, 4291-4303 (2017).
13. Sushkevich V. L. et al. The effect of the active-site structure on the activity of copper mordenite in the aerobic and anaerobic conversion of methane into methanol. *Angew. Chem. Int. Ed. Engl.* **57**, 8906-8910 (2018).
14. Brezicki G. et al. Insights into the Speciation of Cu in the Cu-H-Mordenite Catalyst for the Oxidation of Methane to Methanol. *ACS Catal.* **9**, 5308-5319 (2019).
15. Brezicki G. et al. Effect of the Co-cation on Cu speciation in Cu-exchanged mordenite and ZSM-5 catalysts for the oxidation of methane to methanol. *ACS Catal.* **11**, 4973-4987 (2021).
16. Knorpp A. J. et al. Comparative performance of Cu-zeolites in the isothermal conversion of methane to methanol. *Chem. Commun. (Camb)* **55**, 11794-11797 (2019).
17. Dinh K. T. et al. Continuous partial oxidation of methane to methanol catalyzed by diffusion-paired copper dimers in copper-exchanged zeolites. *J. Am. Chem. Soc.* **141**, 11641-11650 (2019).
18. Koishybay A. et al. Water is the oxygen source for methanol produced in partial oxidation of methane in a flow reactor over Cu-SSZ-13. *J. Am. Chem. Soc.* **142**, 11962-11966 (2020).
19. Knorpp A.J. et al. The influence of zeolite morphology on the conversion of methane to methanol on copper-exchanged omega zeolite (MAZ). *Catal. Sci. Technol.* **9**, 2806-2811 (2019).
20. Dyballa M. et al. Zeolite surface methoxy groups as key intermediates in the stepwise conversion of methane to methanol. *ChemCatChem* **11**, 5022-5026 (2019).
21. Sun L. et al. Water-involved methane-selective catalytic oxidation by dioxygen over copper zeolites. *Chem* **7**, 1557-1568 (2021).
22. Jeong Y. R. et al. Continuous synthesis of methanol from methane and steam over copper-mordenite. *ACS Catal.* **11**, 1065-1070 (2021).
23. Sogukkanli, S. et al. Selective methanol formation via CO-assisted direct partial oxidation of methane over copper-containing CHA-type zeolites prepared by one-pot synthesis. *Green Chem.* **23**, 2148-2154 (2021).
24. Memioglu O. et al. A potential catalyst for continuous methane partial oxidation to methanol using N_2O: Cu-SSZ-39. *Chem. Commun. (Camb)* **57**, 1364-1367 (2021).
25. Ohyama J. et al. Catalytic direct oxidation of methane to methanol by redox of copper mordenite. *Catal. Sci. Technol.* **11**, 3437-3446 (2021).
26. Togo A. et al. First principles phonon calculations in materials science. *Scripta Mater.* **108**, 1-5 (2015).
27. Jangjou Y. et al. Nature of Cu active centers in Cu-SSZ-13 and their responses to SO_2 exposure. *ACS Catal.* **8**, 1325-1337 (2018).
28. Kwak J. H. et al. Following the movement of Cu ions in a SSZ-13 zeolite during dehydration, reduction and adsorption: A combined in situ TP-XRD, XANES/DRIFTS study. *J. Catal.* **314**, 83-93 (2014).
29. Luo J. et al. New insights into Cu/SSZ-13 SCR catalyst acidity. Part I: Nature of acidic sites probed by NH_3 titration. *J. Catal.* **348**, 291-299 (2017).
30. Beale A. M. et al. Recent advances in automotive catalysis for NO_x emission control by small-pore microporous materials. *Chem. Soc. Rev.* **44**, 7371-7405 (2015).
31. Gao F. et al. Current understanding of Cu-exchanged chabazite molecular sieves for use as commercial diesel engine deNO_x catalysts. *Top. Catal.* **56**, 1441-1459 (2013).
32. Gao F. et al. Effects of alkali and alkaline earth cocations on the activity and hydrothermal

stability of Cu/SSZ-13 NH_3–SCR catalysts. *ACS Catal.* **5**, 6780-6791 (2015).
33. Gao F. et al. Effects of Si/Al ratio on Cu/SSZ-13 NH_3-SCR catalysts: Implications for the active Cu species and the roles of Brønsted acidity. *J. Catal.* **331**, 25-38 (2015).
34. Paolucci C. et al. Catalysis in a cage: condition-dependent speciation and dynamics of exchanged Cu cations in SSZ-13 zeolites. *J. Am. Chem. Soc.* **138**, 6028-6048 (2016).
35. Jiang H. et al. Cu/SSZ-13 zeolites prepared by in situ hydrothermal synthesis method as NH_3-SCR catalysts: Influence of the Si/Al ratio on the activity and hydrothermal properties. *Fuel* **255**, (2019).
